# Neighborhood Economic Deprivation, Hispanic Ethnic Enclaves, and Congenital Anomalies in Texas

**DOI:** 10.1002/bdr2.70114

**Published:** 2026-08-31

**Authors:** Jeremy M. Schraw, Rutu A. Rathod, Amy E. Hughes, Sandi L. Pruitt, Russell S. Kirby, Gary M. Shaw, Philip J. Lupo

**Affiliations:** ^1^ Department of Pediatrics Baylor College of Medicine Houston Texas USA; ^2^ Peter O'Donnell Jr. School of Public Health University of Texas Southwestern Medical Center Dallas Texas USA; ^3^ Department of Epidemiology, Fay W Boozman College of Public Health University of Arkansas for Medical Sciences Little Rock Arkansas USA; ^4^ Winthrop P. Rockefeller Cancer Institute University of Arkansas for Medical Sciences Little Rock Arkansas USA; ^5^ Harold C. Simmons Comprehensive Cancer Center University of Texas Southwestern Medical Center Dallas Texas USA; ^6^ Chiles Center, College of Public Health University of South Florida Tampa Florida USA; ^7^ Department of Pediatrics Stanford University Stanford California USA; ^8^ Department of Pediatrics Emory University School of Medicine Atlanta Georgia USA; ^9^ Aflac Cancer and Blood Disorders Center at Children's Healthcare of Atlanta Atlanta Georgia USA

**Keywords:** birth defects, congenital anomalies, epidemiology, pregnancy, social determinants, socioeconomic status, structural determinants

## Abstract

**Background:**

Congenital anomalies are common, costly, and potentially life‐threatening. The impacts of structural determinants of health on risk for these conditions have scarcely been studied. We evaluated associations between residence in economically disadvantaged neighborhoods or Hispanic/Latino enclaves and congenital anomalies.

**Methods:**

We performed a registry linkage study of 7.25 million births and 332,935 cases with anomalies in Texas, 1999–2018. We defined exposure groups using maternal residential address at delivery or termination and tract‐level Yost socioeconomic index and Hispanic/Latino enclave index scores: high neighborhood socioeconomic status (nSES)‐non‐enclave (referent), high nSES‐enclave, low nSES‐non‐enclave, and low nSES‐enclave. We estimated adjusted birth prevalence ratios (aPRs) and 95% confidence intervals (CIs) for 146 congenital anomalies using Poisson regression.

**Results:**

Maternal residence in a low nSES area was associated with increased prevalence of microcephaly, atrial septal defect, pulmonary artery and pulmonary valve anomalies, patent ductus arteriosus, anomalies of the great veins, polydactyly, and Down syndrome (PRs 1.06–1.96) but decreased prevalence of hypospadias, epispadias, and chordee (PRs 0.67–0.89). Maternal residence in an enclave was associated with increased prevalence of ventricular septal defect, pulmonary artery anomalies, female genital anomalies, and Down syndrome (PRs 1.09–1.96). Pyloric stenosis was associated with residence in a low nSES neighborhood (enclave: PR 1.09, CI 1.03–1.14; non‐enclave: PR 1.12, CI 1.06–1.17) and inversely associated with residence in a high nSES enclave (PR 0.89, CI 0.80–0.98).

**Conclusions:**

Neighborhood socioeconomic and sociodemographic factors are associated with congenital anomalies, suggesting differences in risk factors or ascertainment in economically disadvantaged neighborhoods or Hispanic/Latino enclaves.

**Abbreviations**
ACS: American Community Survey
BMI: body mass index
BPA: British Pediatric Association
CHD: congenital heart disease
CI: confidence interval
NBDPS: National Birth Defects Prevention Study
nSES: neighborhood socioeconomic status
NTD: neural tube defect
OR: odds ratio
PR: prevalence ratio
TBDR: Texas Birth Defects Registry
UK: United Kingdom

## 1 Introduction

Congenital anomalies affect ≥ 3% of U.S. births and remain the leading cause of death in infancy (Centers for Disease Control and Prevention 2025). Differences in their prevalence have been described by maternal race, ethnicity, and socioeconomic status. For example, neural tube defects (NTDs) are more common in Hispanic than non‐Hispanic people whereas the opposite appears true for hypospadias, while polydactyly is more common but gastroschisis less common among non‐Hispanic Black than non‐Hispanic White people (Canfield, Honein, et al. 2006; Kucik et al. 2012; Ryan et al. 2019; Schraw et al. 2023). These differences may be partially attributable to social, economic, cultural, and health factors like educational attainment, income, diet, and chronic health conditions (Grewal et al. 2009; Peyvandi et al. 2020; Wasserman et al. 1998; Yang et al. 2008). Most studies evaluating sociodemographic and socioeconomic characteristics in relation to congenital anomalies focused on maternal or household factors like education and income.

Neighborhood characteristics influence pregnancy outcomes through maternal nutrition, health behaviors, chronic health conditions, prenatal care access, opportunities for economic advancement, exposure to environmental toxins, and other mechanisms independent of individual‐ and household‐level factors (Diez Roux 2018; Krieger et al. 1997). The United States National Birth Defects Prevention Study (NBDPS) found that higher Neighborhood Deprivation Index scores were associated with NTDs (odds ratio [OR] for highest versus lowest quartile 1.2, 95% confidence interval [CI] 1.0–1.4) and gastroschisis (OR for highest versus lowest tertile 1.3, CI 1.1–1.6) (Neo et al. 2023; Pruitt Evans et al. 2023). Similarly, population‐based studies in California and Ontario reported statistically significant 18%–34% increases in the OR of congenital heart disease (CHD) in the most versus the least economically disadvantaged areas (Miao et al. 2021; Peyvandi et al. 2020). However, we lack a full understanding of the associations between neighborhood characteristics and anomalies, especially less common phenotypes.

To address this, we performed a comprehensive assessment of the associations between two census tract‐level measurements—the Yost socioeconomic index and Hispanic/Latino (‘Hispanic’) ethnic enclave index—and congenital anomalies (Guan et al. 2025; Shariff‐Marco et al. 2021; Yost et al. 2001). Yost index scores serve as an indicator of economic disadvantage. Enclave index scores reflect social, cultural, political, and economic conditions operating within and around neighborhoods characterized by high concentrations of individuals of the same ethnic origin, high proportions of recent immigrants, linguistically isolated households, and ethnic‐specific businesses. Thus, these measures attempt to summarize, at the neighborhood level, processes and conditions that may be associated with the prevalence or ascertainment of anomalies including (for example) residents' healthcare access and utilization, health behaviors, environmental exposures, and allostatic load. By characterizing the associations of these indices with a broad range of anomalies, we hope to generate hypotheses about the causes of congenital anomalies, identify vulnerable populations that would benefit from prevention efforts, and stimulate research into health disparities.

## 2 Methods

We identified all confirmed cases with congenital anomalies in the Texas Birth Defects Registry (TBDR) for the delivery years 1999–2018, regardless of pregnancy outcome (i.e., termination, stillbirth, or livebirth). Reporting is mandatory for fetal deaths > 20 weeks or > 350 g. A detailed evaluation of TBDR practices has been published (Miller 2006). Briefly, it is a population‐based, active surveillance system that collects data on all cases: (1) diagnosed with eligible conditions prenatally or within 1 year of delivery; and (2) delivered by a person residing in Texas. Records are subject to extensive quality checks, including review by clinical geneticists. For our purposes, cases had one or more confirmed six‐digit Centers for Disease Control and Prevention‐modified British Pediatric Association (BPA) diagnostic codes in the 740–759 range. Approximately 5% of anomalies could not be independently verified by registry staff and were excluded; this proportion did not differ between the neighborhood categories defined for our study. To facilitate analysis, we collapsed six‐digit BPA codes to the first four digits, with certain exceptions: (1) we combined spina bifida with/without hydrocephaly (assigned code 741); (2) we combined cleft lip alone and cleft lip with cleft palate (749.1); and (3) we evaluated gastroschisis (756.70) and omphalocele (756.71) separately due to differences in their epidemiology. Lastly, we computed a dummy code (888.8) indicating diagnosis of any monitored anomaly. Linkage to birth and fetal death records was performed to determine birth prevalence denominators and obtain information on sex, maternal residential address at delivery or termination, age, race and ethnicity, educational attainment, previous livebirths, and (after 2004, when Texas adopted the 2003 standard US birth certificate) (National Center for Health Statistics 2023) pre‐pregnancy body mass index (BMI) for all cases and livebirths during the study period. Linkage was successful for > 95% of cases in TBDR; cases that did not link to a birth or fetal death record were excluded from our analyses.

We computed Hispanic ethnic enclave index scores by principal components analysis of Census Bureau data on the percentage of residents in each census tract who were: Hispanic; foreign‐born; Spanish‐speaking with limited English proficiency; or Spanish‐speaking and in linguistically isolated households (Guan et al. 2025; Shariff‐Marco et al. 2021). We chose the Hispanic ethnic enclave index because Hispanic people are the largest minoritized group in Texas, tend to reside in neighborhoods with lower socioeconomic status, and have a higher prevalence of certain congenital anomalies (Canfield, Honein, et al. 2006; Kucik et al. 2012; Ryan et al. 2019; Schraw et al. 2023). For delivery years 1999–2004, we calculated scores using 2000 decennial Census data and geographies; for delivery years 2005–2018, we used 2010 decennial census geographies and 2008–2012 American Community Survey (ACS) data. At the time of analysis, these were the only data available for this novel measure.

The Yost socioeconomic index is a weighted estimate of neighborhood‐level socioeconomic status (nSES) based on principal components analysis of area‐level household income, poverty, rent, home value, employment, education, and occupation (Yost et al. 2001). While no single measure perfectly represents neighborhood socioeconomic context, the Yost index possesses face validity and is transparent, reproducible, and adaptable to a range of geographies and years (Boscoe et al. 2021). For delivery years 1999–2004, we calculated scores using data and geographies from the 2000 decennial census. For delivery years 2005–2009, we used 2006–2010 ACS estimates; for delivery years 2010–2013, we used ACS 2010–2014 estimates; and for delivery years 2014–2018, we used 2013–2017 ACS estimates.

**FIGURE 1:**
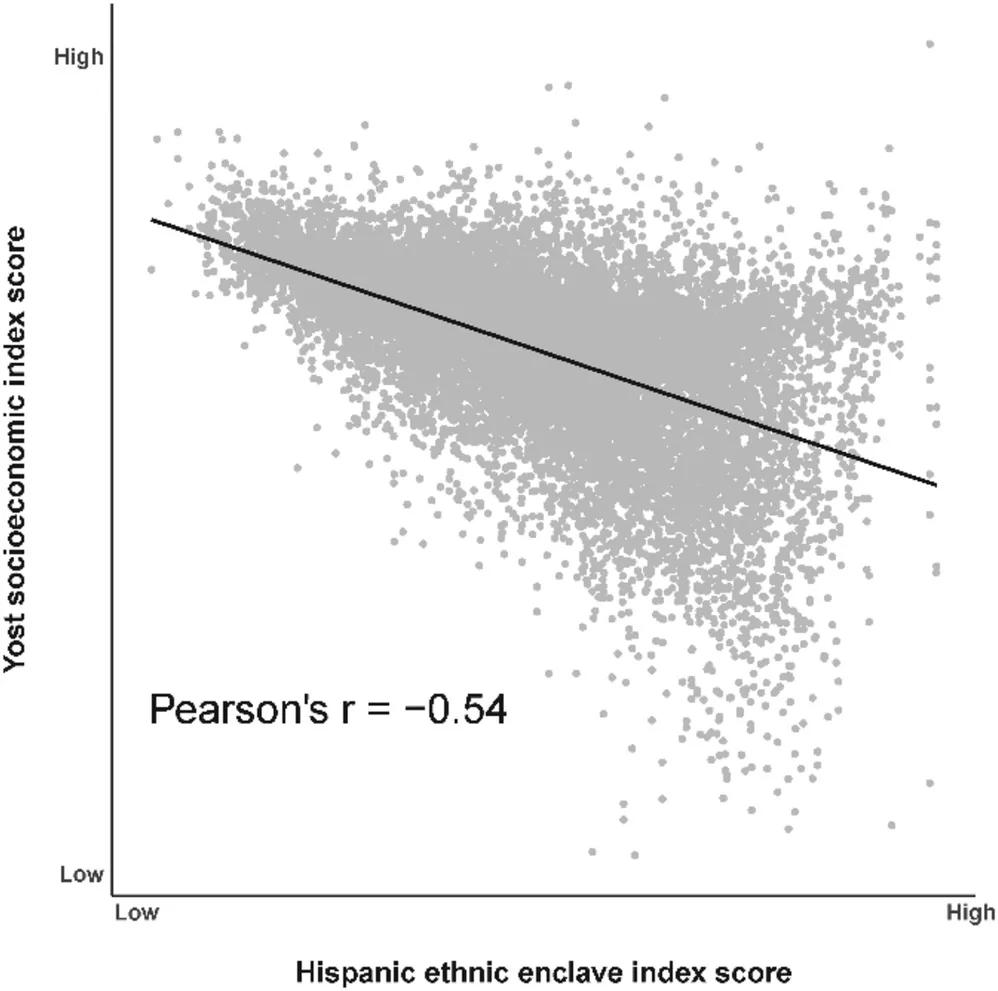
Correlation between census tract‐level Yost socioeconomic index and Hispanic/Latino ethnic enclave index scores, Texas, 1999–2018.

We binned tracts into period‐specific quintiles for each index based on the statewide distribution (Table S1). We classified tracts as enclaves (culturally/ethnically distinct) if they met the following criteria: (1) quintile 5 with > 250 Hispanic residents; or (2) quintile 4 with > 250 Hispanic residents and spatially adjacent to a quintile 5 tract. Greater Yost scores corresponded to higher nSES, and we defined low nSES tracts as those in the lower three quintiles. Because Yost and Hispanic ethnic enclave index scores were correlated (Figure 1), we created a joint exposure variable with mutually exclusive categories: high nSES‐non‐enclave (referent), high nSES‐enclave, low nSES‐non‐enclave, and low nSES‐enclave.

Analyses were conducted using R 4.3.1 (R Core Team, Vienna, Austria). We summarized sample characteristics using counts and percentages. We performed Poisson regression to estimate the prevalence ratio (PR) of each anomaly (*k* = 146), adjusting for the following covariates, selected a priori based on literature review: maternal age (< 20, 20–24, 25–29, 30–34, 35–39, ≥ 40 years), education (no high school diploma, high school diploma or equivalent, higher education or vocational training), previous livebirths (none, one, two, three or more), pre‐pregnancy BMI (< 18.5, 18.5–24.9, 25–29.9, ≥ 30 kg/m^2^), and year of delivery. We computed robust standard errors accounting for clustering of observations by tract (Zeileis et al. 2020). Due to the number of anomalies included in our assessment, we employed a two‐stage approach with multiple testing correction. Prior to analysis, we randomly divided the data into discovery (60%) and replication (40%) partitions. We first evaluated each anomaly in the discovery partition; if prevalence was statistically significantly different from the referent group for at least one neighborhood category at a Bonferroni‐adjusted *p* < 1.14 × 10^−4^ (nominal *p* < 0.05 divided by the number of anomalies), it was reassessed in the replication partition. Those that were statistically significant at *p* < 0.05 and for which the direction of the effect estimate was the same were considered to have replicated. In the text, we focus on replicated associations and present PRs and CIs from the pooled dataset to maximize precision.

We computed a second set of models further adjusted for maternal race (Black, White, additional groups [combined due to low numbers], unknown) and ethnicity (Hispanic/Latina, non‐Hispanic/Latina), which we considered proxies for discriminatory and sociocultural factors influencing exposures and outcomes (Swilley‐Martinez et al. 2023). By comparing models with and without adjustment for race and ethnicity, we sought to understand whether these influenced our findings. We performed subgroup analyses of: (1) offspring delivered by Hispanic people, the largest ethnic group in our study, because the adverse effects of neighborhood factors on pregnancy outcomes may vary by race and ethnicity (Ncube et al. 2016); (2) nulliparous people, because we could not account for potential correlations between infants and fetuses delivered by the same person; and (3) cases with severe microcephaly (head circumference < 3rd percentile for sex and gestational age), as these may be ascertained more consistently and completely.

This study complied with the principles of the Declaration of Helsinki and all relevant laws. It was approved by the Institutional Review Boards of Baylor College of Medicine (H‐31777, approved 9/27/2012) and the Texas Department of State Health Services (18‐046‐BCM, approved 6/12/2023) with a waiver of informed consent.

## 3 Results

**FIGURE 2:**
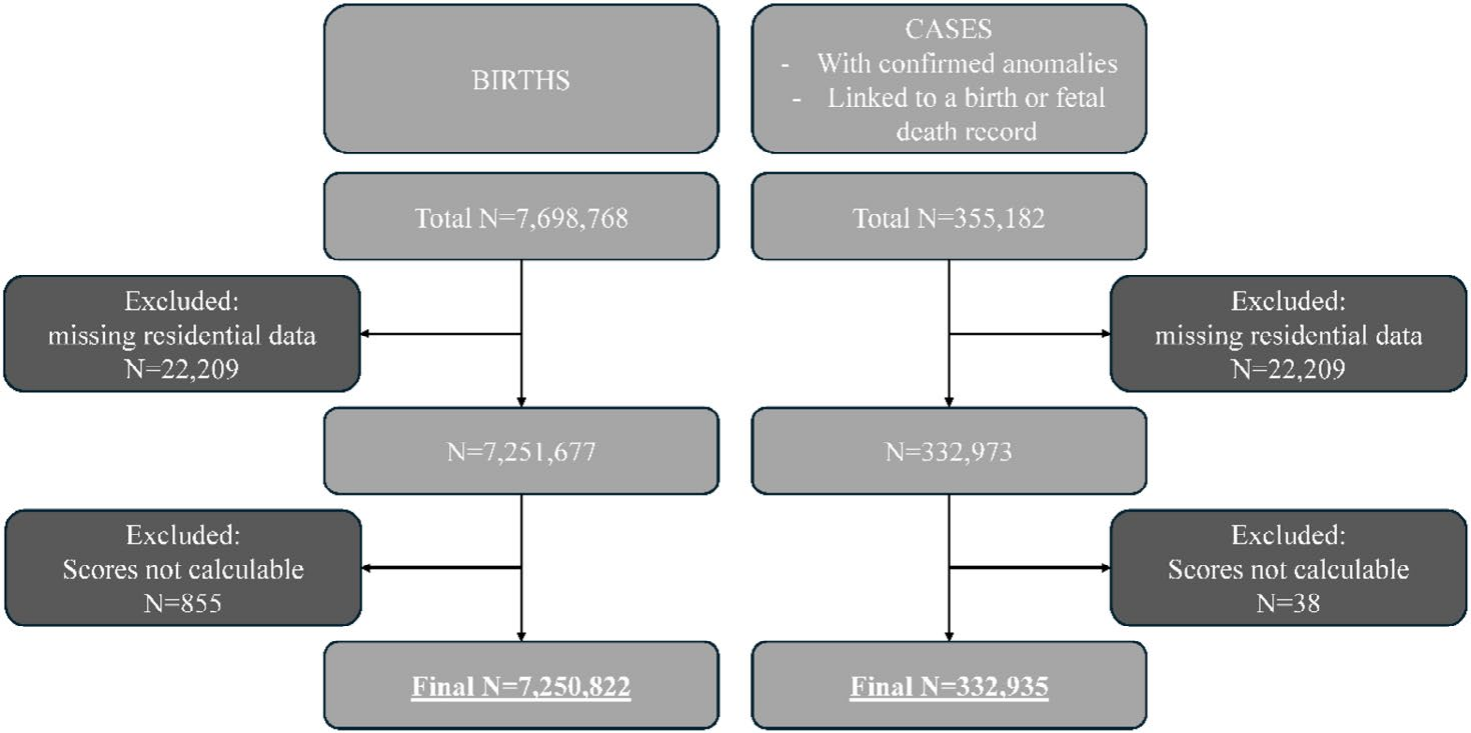
CONSORT diagram describing the number excluded for missing or invalid residential data.

Data on neighborhood characteristics were available for 93.7% of cases and 94.2% of births (Figure 2). Table 1 summarizes characteristics of cases and births. Consistent with published reports (Lary and Paulozzi 2001), a greater proportion of cases were male, and a greater proportion of cases were delivered during the latter half of the study period. People who delivered cases were somewhat more likely to be nulliparous, ≥ 35 years of age, and overweight or obese. Most subjects were delivered by people in high nSES non‐enclaves or low nSES enclaves; fewer were delivered by people in high nSES enclaves. The characteristics of subjects with and without complete residential information are shown in Table S2.

**TABLE 1:** Reproductive and demographic characteristics of people who delivered livebirths and cases with congenital anomalies, Texas, 1999–2018.

	Livebirths, no. (%)	Cases, no. (%)
Total	7,250,822 (100.0)	332,935 (100.0)
Sex		
Male	3,705,613 (51.1)	200,141 (60.2)
Female	3,545,209 (48.9)	132,581 (39.8)
Unknown	0 (0.0)	213 (0.0)
Year of delivery		
1999–2004	1,981,292 (27.3)	66,887 (20.1)
2005–2009	1,880,953 (25.9)	78,816 (23.7)
2010–2014	1,858,540 (25.6)	95,143 (28.6)
2015–2018	1,530,037 (21.1)	92,089 (27.7)
Neighborhood category		
Low nSES‐enclave	2,499,613 (34.5)	114,924 (34.5)
Low nSES‐non‐enclave	1,854,462 (25.6)	84,875 (25.5)
High nSES‐enclave	335,674 (4.6)	14,506 (4.4)
High nSES‐non‐enclave	2,561,073 (35.3)	118,630 (35.6)
Maternal race and ethnicity		
Hispanic/Latina	3,476,679 (47.9)	157,021 (47.2)
Non‐Hispanic Black	848,887 (11.7)	39,935 (12.0)
Non‐Hispanic White	2,535,431 (35.0)	119,279 (35.8)
Additional non‐Hispanic groups	381,533 (5.3)	16,660 (5.0)
Unknown	8272 (0.1)	40 (0.0)
Maternal age (years)		
< 20	842,889 (11.6)	35,981 (10.8)
20–24	1,893,220 (26.1)	81,367 (24.4)
25–29	1,999,062 (27.6)	88,262 (26.5)
30–34	1,607,008 (22.2)	75,417 (22.7)
35–39	745,108 (10.3)	40,459 (12.2)
≥ 40	163,183 (2.3)	11,359 (3.4)
Unknown	352 (0.0)	0 (0.0)
Maternal education		
No high school diploma	1,855,327 (25.6)	81,614 (24.5)
High school diploma or equivalent	2,015,116 (27.8)	94,700 (28.4)
Any post‐high school	3,339,842 (46.1)	154,880 (46.5)
Unknown	40,537 (0.6)	1741 (0.5)
Number of previous livebirths		
0	2,753,218 (38.0)	132,759 (39.9)
1	2,233,977 (30.8)	96,849 (29.1)
2	1,295,873 (17.9)	57,631 (17.3)
≥ 3	885,393 (12.2)	42,825 (12.9)
Unknown	82,361 (1.1)	2871 (0.9)
Pre‐pregnancy BMI category^a^		
Underweight (< 18.5)	207,113 (2.9)	10,033 (3.0)
Normal weight (18.5–24.9)	2,451,992 (33.8)	118,424 (35.6)
Overweight (25–29.9)	1,336,470 (18.4)	65,875 (19.8)
Obese (≥ 30)	1,240,651 (17.1)	69,197 (20.8)
Unknown	2,014,596 (27.8)	69,406 (20.8)

Abbreviations: BMI = body mass index; nSES = neighborhood socioeconomic status.

^a^ Data on maternal pre‐pregnancy body mass index were not collected prior to 2005. BMI was classified as unknown among people who delivered a case or livebirth between January 1, 1999–December 31, 2004.

In discovery analyses, tract‐level characteristics were associated with any monitored anomaly and 27 central nervous system, craniofacial, cardiac, gastrointestinal, genitourinary, musculoskeletal, and chromosomal anomalies after Bonferroni correction (Table S3). Among these (Table S4), 25 showed similar associations (same direction of effect and *p* < 0.05) in replication analyses.

**FIGURE 3:**
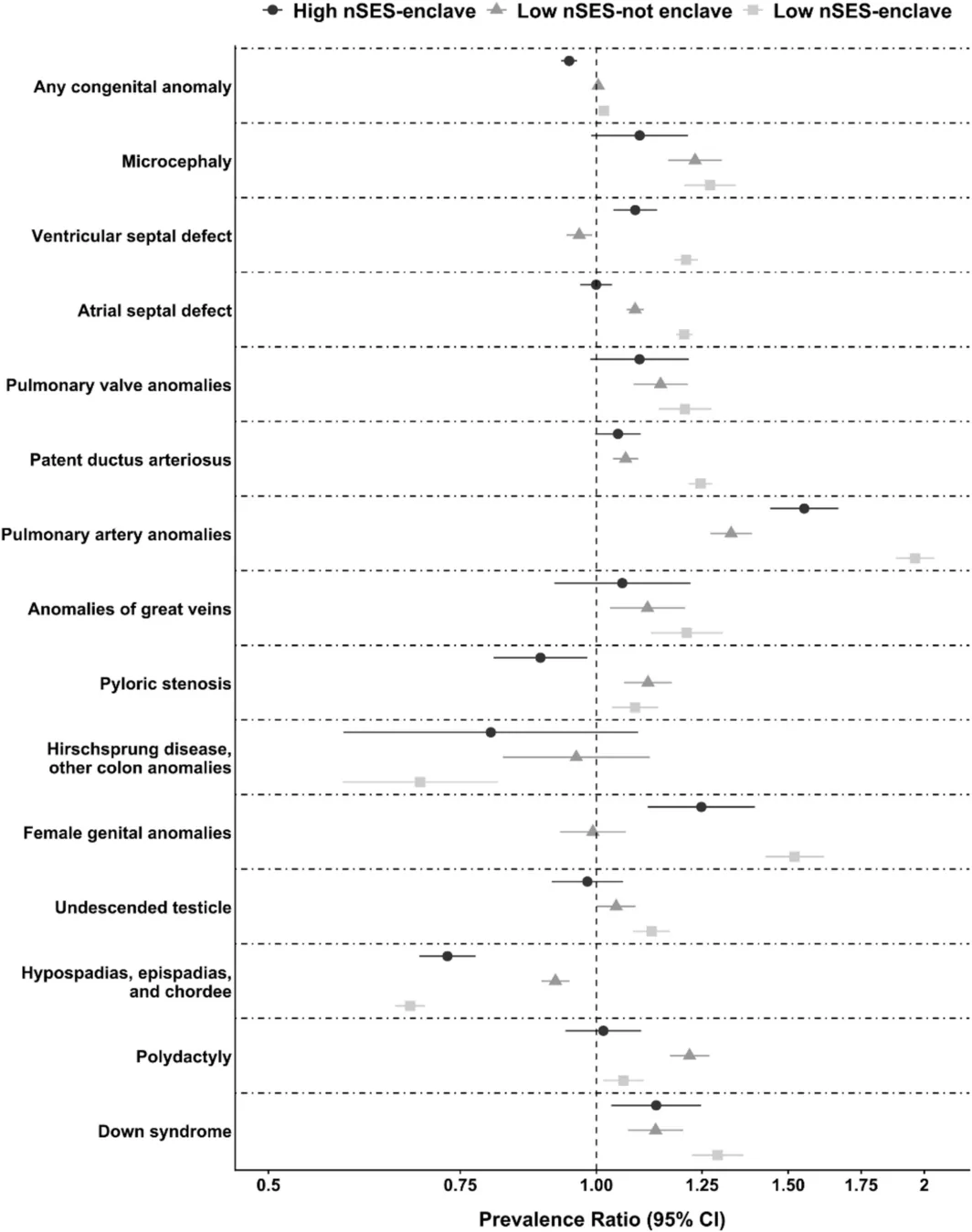
Neighborhood socioeconomic status, Hispanic/Latino ethnic enclave status, and selected congenital anomalies. *X*‐axis displays the prevalence ratio and 95% confidence interval relative to non‐enclaves with high socioeconomic status. Models are adjusted for year of delivery and maternal age, educational attainment, previous livebirths, and body mass index.

We observed increased prevalence of several anomalies in low nSES enclaves (Table 2, Figure 3), including microcephaly (PR 1.27, CI 1.20–1.34), ventricular septal defect (PR 1.21, CI 1.18–1.24), atrial septal defect (PR 1.20, CI 1.18–1.22), pulmonary valve anomalies (PR 1.21, CI 1.14–1.27), patent ductus arteriosus (PR 1.25, CI 1.22–1.28), pulmonary artery anomalies (PR 1.96, CI 1.88–2.04), anomalies of the great veins (PR 1.21, CI 1.12–1.31), female genital anomalies (PR 1.52, CI 1.43–1.62), undescended testicle (PR 1.12, CI 1.08–1.17), polydactyly (PR 1.06, 1.01–1.10), and Down syndrome (PR 1.29, CI 1.22–1.36). Conversely, we observed decreased prevalence of Hirschsprung disease (PR 0.69, CI 0.58–0.81), and hypospadias, epispadias, and chordee (PR 0.67, CI 0.65–0.70). Among people in high nSES enclaves, we observed differences in the prevalence of pulmonary artery anomalies (PR 1.55, CI 1.44–1.67), female genital anomalies (PR 1.25, CI 1.11–1.40), hypospadias, epispadias and chordee (PR 0.73, CI 0.69–0.77), and Down syndrome (PR 1.13, CI 1.03–1.25). In low nSES non‐enclaves, we observed increases in microcephaly (PR 1.23, CI 1.16–1.30), atrial septal defect (PR 1.09, CI 1.07–1.11), pulmonary valve anomalies (PR 1.15, CI 1.08–1.21), patent ductus arteriosus (PR 1.06, CI 1.04–1.09), pulmonary artery anomalies (PR 1.33, CI 1.27–1.39), polydactyly (PR 1.22, CI 1.17–1.27), and Down syndrome (PR 1.13, CI 1.07–1.20). Conversely, we observed modestly decreased prevalence of ventricular septal defect (PR 0.96, CI 0.94–0.99). Prevalence of pyloric stenosis was increased in low nSES enclaves (PR 1.09, CI 1.03–1.14) but decreased in high nSES enclaves (PR 0.89, CI 0.80–0.98).

**TABLE 2:** Associations of selected congenital anomalies with residence in a Hispanic/Latino ethnic enclave and/or neighborhood with low socioeconomic status, Texas, 1999–2018.

	Cases, no.	Prevalence^a^	aPR (95% CI)^b^	aPR (95% CI)^c^
Any monitored congenital anomaly				
High nSES‐non‐enclave	118,630	463.2	1.00	1.00
High nSES‐enclave	14,506	432.1	0.94 (0.93–0.96)	0.96 (0.95–0.98)
Low nSES‐non‐enclave	84,875	457.7	1.00 (0.99–1.01)	1.00 (0.99–1.01)
Low nSES‐enclave	114,924	459.8	1.02 (1.01–1.03)	1.04 (1.03–1.05)
Microcephaly				
High nSES‐non‐enclave	2760	10.8	1.00	1.00
High nSES‐enclave	425	12.7	1.10 (0.99–1.21)	1.07 (0.97–1.19)
Low nSES‐non‐enclave	2725	14.7	1.23 (1.16–1.30)	1.18 (1.12–1.25)
Low nSES‐enclave	4001	16	1.27 (1.20–1.34)	1.23 (1.16–1.30)
Ventricular septal defect				
High nSES‐non‐enclave	14,421	56.3	1.00	1.00
High nSES‐enclave	2057	61.3	1.09 (1.04–1.14)	1.04 (0.99–1.09)
Low nSES‐non‐enclave	9885	53.3	0.96 (0.94–0.99)	0.96 (0.94–0.99)
Low nSES‐enclave	17,101	68.4	1.21 (1.18–1.24)	1.14 (1.11–1.17)
Atrial septal defect				
High nSES‐non‐enclave	29,322	114.5	1.00	1.00
High nSES‐enclave	3823	113.9	1.00 (0.97–1.03)	0.98 (0.95–1.01)
Low nSES‐non‐enclave	23,215	125.2	1.09 (1.07–1.11)	1.08 (1.06–1.10)
Low nSES‐enclave	34,869	139.5	1.20 (1.18–1.22)	1.17 (1.15–1.19)
Pulmonary valve anomalies				
High nSES‐non‐enclave	2843	11.1	1.00	1.00
High nSES‐enclave	412	12.3	1.10 (0.99–1.22)	1.10 (0.99–1.22)
Low nSES‐non‐enclave	2437	13.1	1.15 (1.08–1.21)	1.13 (1.07–1.19)
Low nSES‐enclave	3507	14	1.21 (1.14–1.27)	1.20 (1.13–1.27)
Patent ductus arteriosus				
High nSES‐non‐enclave	14,057	54.9	1.00	1.00
High nSES‐enclave	1914	57	1.05 (1.00–1.10)	1.01 (0.97–1.07)
Low nSES‐non‐enclave	10,529	56.8	1.06 (1.04–1.09)	1.05 (1.03–1.08)
Low nSES‐enclave	16,869	67.5	1.25 (1.22–1.28)	1.20 (1.16–1.23)
Pulmonary artery anomalies				
High nSES‐non‐enclave	4411	17.2	1.00	1.00
High nSES‐enclave	902	26.9	1.55 (1.44–1.67)	1.36 (1.27–1.47)
Low nSES‐non‐enclave	4446	24	1.33 (1.27–1.39)	1.29 (1.23–1.35)
Low nSES‐enclave	8904	35.6	1.96 (1.88–2.04)	1.66 (1.59–1.73)
Aortic valve insufficiency				
High nSES‐non‐enclave	2033	7.9	1.00	1.00
High nSES‐enclave	242	7.2	0.90 (0.79–1.03)	0.90 (0.79–1.04)
Low nSES‐non‐enclave	1659	8.9	1.11 (1.04–1.19)	1.12 (1.05–1.20)
Low nSES‐enclave	2190	8.8	1.07 (1.00–1.15)	1.07 (1.00–1.15)
Anomalies of the great veins				
High nSES‐non‐enclave	1514	5.9	1.00	1.00
High nSES‐enclave	212	6.3	1.06 (0.91–1.22)	1.01 (0.87–1.17)
Low nSES‐non‐enclave	1238	6.7	1.11 (1.03–1.21)	1.10 (1.02–1.20)
Low nSES‐enclave	1841	7.4	1.21 (1.12–1.31)	1.14 (1.05–1.24)
Pyloric stenosis				
High nSES‐non‐enclave	3588	14	1.00	1.00
High nSES‐enclave	442	13.2	0.89 (0.80–0.98)	0.89 (0.81–0.99)
Low nSES‐non‐enclave	3292	17.8	1.12 (1.06–1.17)	1.16 (1.10–1.22)
Low nSES‐enclave	4408	17.6	1.09 (1.03–1.14)	1.09 (1.04–1.15)
Hirschsprung disease				
High nSES‐non‐enclave	426	1.7	1.00	1.00
High nSES‐enclave	44	1.3	0.80 (0.59–1.09)	0.97 (0.71–1.32)
Low nSES‐non‐enclave	298	1.6	0.96 (0.82–1.12)	0.95 (0.81–1.11)
Low nSES‐enclave	283	1.1	0.69 (0.58–0.81)	0.89 (0.74–1.06)
Female genital anomalies				
High nSES‐non‐enclave	2062	8.1	1.00	1.00
High nSES‐enclave	352	10.5	1.25 (1.11–1.40)	1.07 (0.95–1.20)
Low nSES‐non‐enclave	1529	8.2	0.99 (0.93–1.06)	0.95 (0.89–1.02)
Low nSES‐enclave	3293	13.2	1.52 (1.43–1.62)	1.25 (1.17–1.34)
Undescended testicle				
High nSES‐non‐enclave	5935	23.2	1.00	1.00
High nSES‐enclave	762	22.7	0.98 (0.91–1.06)	0.97 (0.90–1.05)
Low nSES‐non‐enclave	4438	23.9	1.04 (1.00–1.09)	1.02 (0.98–1.07)
Low nSES‐enclave	6448	25.8	1.12 (1.08–1.17)	1.11 (1.06–1.16)
Hypospadias, epispadias, chordee				
High nSES‐non‐enclave	13,380	52.2	1.00	1.00
High nSES‐enclave	1188	35.4	0.73 (0.69–0.77)	0.85 (0.80–0.91)
Low nSES‐non‐enclave	7839	42.3	0.92 (0.89–0.94)	0.92 (0.90–0.95)
Low nSES‐enclave	7159	28.6	0.67 (0.65–0.70)	0.84 (0.81–0.87)
Polydactyly				
High nSES‐non‐enclave	5194	20.3	1.00	1.00
High nSES‐enclave	682	20.3	1.01 (0.94–1.10)	1.04 (0.96–1.13)
Low nSES‐non‐enclave	4767	25.7	1.22 (1.17–1.27)	1.04 (0.99–1.08)
Low nSES‐enclave	5395	21.6	1.06 (1.01–1.10)	1.06 (1.01–1.11)
Down syndrome				
High nSES‐non‐enclave	3380	13.2	1.00	1.00
High nSES‐enclave	490	14.6	1.13 (1.03–1.25)	1.05 (0.95–1.15)
Low nSES‐non‐enclave	2116	11.4	1.13 (1.07–1.20)	1.12 (1.05–1.18)
Low nSES‐enclave	3643	14.6	1.29 (1.22–1.36)	1.16 (1.10–1.23)

Abbreviations: aPR = adjusted prevalence ratio; CI = 95% confidence interval.

^a^ Per 10,000 live births.

^b^ Adjusted for year of delivery, maternal age (< 20, 20–24, 25–29, 30–34, 35–39, ≥ 40 years), education (no high school diploma or equivalent, high school diploma or equivalent, any higher education or vocational training), number of previous livebirths (none, one, two, three or more), and pre‐pregnancy body mass index category (< 18.5, 18.5–24.9, 25–29.9, ≥ 30, unknown). Relative to a referent category of individuals residing in high nSES‐non‐enclave neighborhoods.

^c^ Adjusted for the variables in (b), plus maternal race (Black, White, other, or unknown) and ethnicity (Hispanic/Latina or non‐Hispanic/Latina).

**FIGURE 4:**
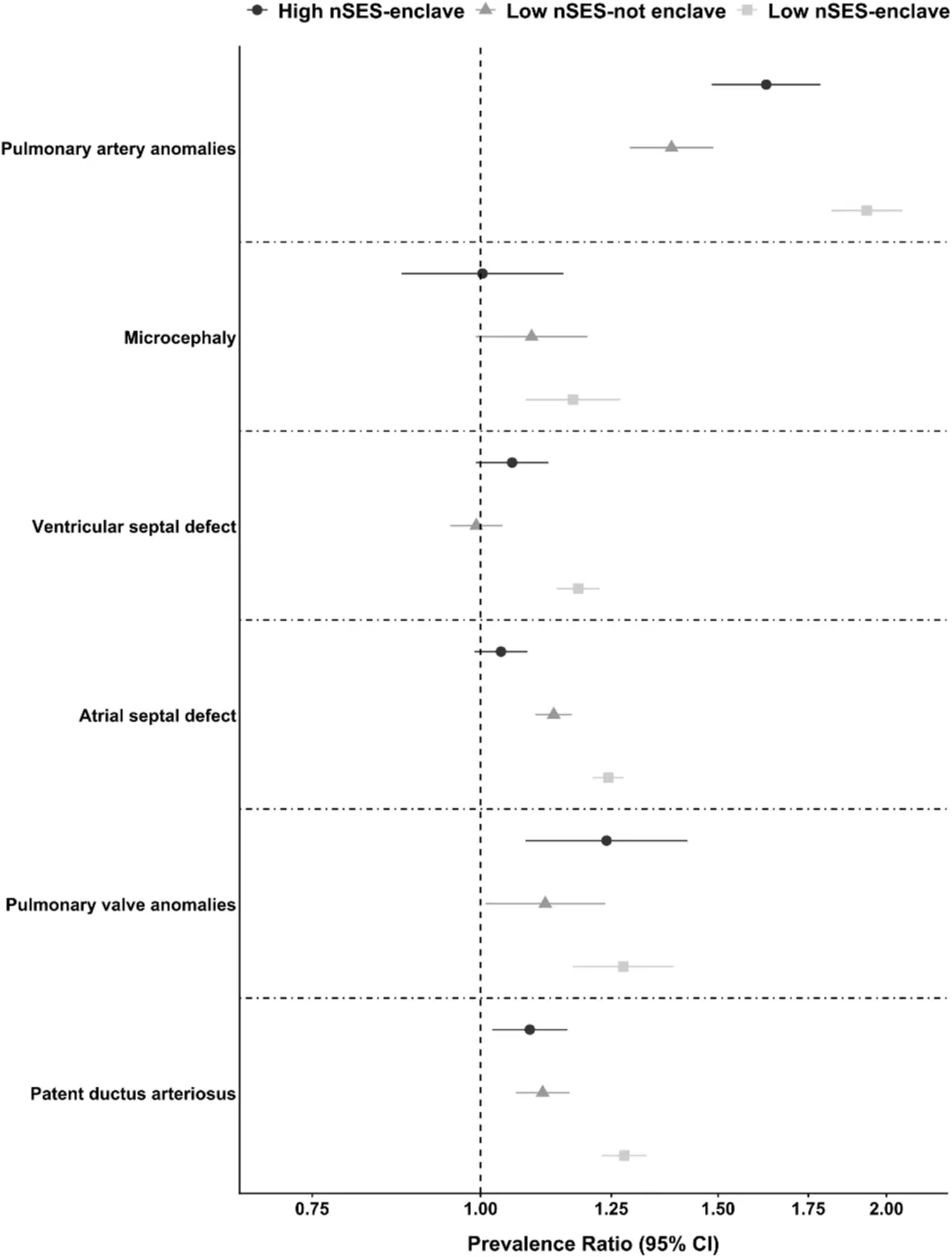
Neighborhood socioeconomic status, Hispanic/Latino ethnic enclave status, and selected congenital anomalies among Hispanic/Latino people. *X*‐axis displays the prevalence ratio and 95% confidence interval of each anomaly relative to non‐enclaves with high socioeconomic status. Models are adjusted for year of delivery and maternal age, educational attainment, previous livebirths, and body mass index.

Associations between neighborhood characteristics, Hirschsprung disease, female genital anomalies, and polydactyly were not statistically significant after adjustment for maternal race and ethnicity. Most other associations (e.g., pulmonary artery anomalies, microcephaly) were modestly attenuated (Table 2). Neighborhood characteristics were associated with any monitored anomaly and ten specific anomalies among Hispanic people, similar to the primary analysis (Figure 4, Table S5). Microcephaly, pyloric stenosis, and female genital anomalies were associated with neighborhood characteristics in the total population but did not reach statistical significance in this subgroup analysis. Analyses restricted to cases with severe microcephaly and subjects delivered by nulliparous women were substantially similar to those from the overall analysis and are presented in Tables S6 and S7, respectively.

## 4 Discussion

Congenital anomalies are costly and potentially life‐threatening adverse pregnancy outcomes (Centers for Disease Control and Prevention 2025). The impact of neighborhood environment on risk for these conditions is largely unknown. Here, we demonstrate that residence in a Hispanic ethnic enclave or neighborhood with low socioeconomic status is associated with several major anomalies in Texas after adjusting for maternal factors. Conditions in economically disadvantaged neighborhoods (pollution; poor walkability; limited access to healthy food options, recreational spaces, and health services) may contribute to chronic disease and inadequate prenatal care, increasing the risk of an affected pregnancy. Conversely, residents may face challenges obtaining diagnoses due to reduced healthcare access, causing incomplete ascertainment. As many Hispanic ethnic enclaves in Texas are economically disadvantaged, these mechanisms may, in part, mediate their proposed health effects. Additionally, however, it is important to consider their unique cultural context. Whereas social cohesion may ameliorate the health effects of adverse structural environments (Shariff‐Marco et al. 2020), other characteristics discussed below could be linked to increased prevalence or reduced ascertainment of certain anomalies. Although our study cannot identify specific factors driving the observed differences, it can inform research into health disparities and assist in identifying vulnerable populations.

No study has evaluated the risk of delivering a child or fetus with a congenital anomaly among people living in enclaves. We observed increased prevalence of several anomalies in low nSES but not high nSES enclaves, with some exceptions (increased prevalence of pulmonary artery anomalies and Down syndrome, decreased prevalence of hypospadias and Hirschsprung disease). Some of these findings may reflect disparities in healthcare access and utilization among residents. For example, as hypospadias may be diagnosed at the time of circumcision (Keays and Dave 2017), and as circumcision is less prevalent in Hispanic compared to non‐Hispanic boys (Jacobson et al. 2021; Morris et al. 2014), delayed diagnosis may explain the apparently lower prevalence of hypospadias among boys in enclaves. Similarly, in the context of work demonstrating that low maternal socioeconomic status is associated with the risk of a Down syndrome‐affected pregnancy (Christianson et al. 2004; Hunter et al. 2013; Torfs and Christianson 2003), the greater prevalence of Down syndrome among children or fetuses in enclaves may reflect reduced access to or uptake of prenatal screening (Hui et al. 2018; Kuppermann et al. 2006). This is because early losses (≤ 20 gestational weeks), those that were medically managed, and those that occurred outside the facilities where the Registry conducted surveillance may not have been reported. Conversely, reduced access to prenatal testing among women in low nSES areas or Hispanic ethnic enclaves may have resulted in a greater number of live births with Down syndrome. An alternative or complementary explanation is that greater pre‐pregnancy exposure to harmful environmental agents among people in low SES neighborhoods or enclaves could lead to meiotic defects including Down syndrome (Nagaoka et al. 2012). Regardless, the increasing prevalence of Down syndrome among Black and Hispanic individuals underscores the public health importance of our finding (Chaiken et al. 2022). Of note, the typically stronger associations among women in low nSES compared to high nSES enclaves suggest that these challenges are compounded by material hardship. Furthermore, the observation that associations were often attenuated by adjustment for maternal race and ethnicity suggests that discriminatory and sociocultural factors linked to self‐reported race and ethnicity contribute to but do not fully explain the observed differences. While concerning, our findings provide actionable information regarding important health disparities.

More work has been done to understand the effects of neighborhood socioeconomic characteristics on congenital anomalies. Miao et al. investigated neighborhood disadvantage and CHD among births and late‐stage terminations in Ontario (Miao et al. 2021). For dissemination areas (400–700 residents on average) in the highest quintile of most deprivation measures, the authors observed 10%–30% increases in the OR of CHD. An earlier registry linkage study, also performed in Ontario, demonstrated a 20% difference (CI 15%–24%) in the prevalence of CHD between extremes of neighborhood income and a 27% difference (CI 21%–31%) with respect to percent residents with university degrees (Agha et al. 2011). A California case–control study found no association between block group‐level SES and conotruncal defects (Tetralogy of Fallot and d‐transposition of the great arteries) (Carmichael et al. 2009). Multicenter case–control studies from the United Kingdom (UK) and Latin America reported significant increases in the risk of cardiac septal malformations in low nSES areas (UK: OR 2.82, CI 1.43–5.56; Latin America: OR 1.38, *p* < 0.01) (Pawluk et al. 2014; Vrijheid et al. 2000). The UK study reported non‐statistically significant increases for all cardiac anomalies (OR 1.59, CI 0.98–2.59) and malformations of the cardiac valves (OR 1.49, CI 0.66–3.36). Our findings are consistent with these, save that effects estimated in the U.K. study were stronger.

Population‐based studies in the United States and Canada reported 20%–30% increases in NTDs among populations in neighborhoods with low SES or education (Agha et al. 2013; Pruitt Evans et al. 2023). A study using data from the California Birth Defects Monitoring Program found larger 30%–80% increases in NTD risk for neighborhoods with high rates of poverty, rental housing, and crowding (Wasserman et al. 1998). In contrast, subsequent population‐based case–control studies conducted in California and the UK identified no consistent associations between neighborhood socioeconomic indicators and NTDs (Grewal et al. 2009; Vrijheid et al. 2000). Like these, we observed no statistically significant associations of tract‐level characteristics with NTDs. However, in our discovery analysis, we reported nominally significant increases in the prevalence of spina bifida in low nSES enclaves (PR 1.27, CI 1.11–1.46) and low nSES non‐enclaves (PR 1.19, CI 1.03–1.37) that did not meet our pre‐specified criteria for multiple testing‐corrected statistical significance. Given the stringency of our multiple testing correction, it is possible these are false negative findings. Differences in dietary norms and health behaviors, such as consumption of corn masa flour and tortillas (for which folic acid fortification is not mandatory) and reduced rates of folic acid supplementation provide plausible mechanisms of association (Canfield, Przybyla, et al. 2006; Kancherla et al. 2019).

Other anomalies are less well studied. With respect to orofacial clefts, four studies reported associations between low nSES and cleft lip (Clark et al. 2003; Durning et al. 2007; Lupo et al. 2015; Pawluk et al. 2014), while two found none (Carmichael et al. 2009, 2003). Our own findings provide no evidence of an association. We did, however, observe associations with pyloric stenosis and genitourinary anomalies. An inverse association between tract‐level median household income and pyloric stenosis has been described, and rural residence has been evaluated as a potential risk factor (Mowrer et al. 2019; To and others 2005). Hypospadias was associated with neighborhood deprivation in a nationwide Swedish study, but not in a multicenter case–control study from Latin America (Li et al. 2019; Pawluk et al. 2014).

Our study included > 330,000 cases ascertained by a population‐based, active birth defects surveillance system and a referent group of 7.25 million livebirths. Given its design, it should be free from selection bias, and the large sample enabled us to evaluate a broad range of phenotypes, apply a stringent multiple testing correction, and conduct replication analyses, techniques that are uncommon among epidemiologic studies of anomalies. Perhaps most importantly, our investigation focuses on neighborhood‐level exposures that are collectively experienced by everyone, and our emphasis on Hispanic ethnic enclaves is novel. At the same time, we are mindful of certain limitations. Birth and fetal death certificates used in this study did not list address at conception, so we may have misclassified women who moved during pregnancy. We previously demonstrated that residential mobility during pregnancy was comparable among mothers of cases with NTDs and mothers of infants without anomalies in the NBDPS, and that estimated benzene exposure was similar for address at delivery and address at conception (Lupo et al. 2010). Therefore, exposure misclassification is likely minimal and non‐differential. Secondly, we were unable to account for potential confounders that were missing from vital records (e.g., maternal prenatal vitamin or folic acid consumption, occupation) or for which these have poor sensitivity (e.g., alcohol and tobacco consumption) (Howland et al. 2015; Ziogas et al. 2022). We used data from the 2000 decennial census and 2008–2012 ACS to identify Hispanic ethnic enclaves. Because enclave status may change over time, our approach may have misclassified some deliveries. However, we recently demonstrated that 88%–92% of census tracts retained their designation as Asian American enclaves based on 2008–2012 and 2015–2019 ACS data in a pooled analysis of five states including Texas (Canchola et al. 2026), suggesting that ethnic enclaves are generally stable across time.

Congenital anomalies are common and remain the leading cause of death during infancy. The potential contributions of neighborhood social and economic factors to these conditions have not been investigated comprehensively. We present evidence that neighborhood socioeconomic deprivation and residence in a Hispanic ethnic enclave are associated with the prevalence of several major anomalies in Texas, one of the largest and most diverse US states. Our findings suggest that the social environment influences both risk for and ascertainment of anomalies and identify targets for interventions to increase health equity.

## Author Contributions

**Jeremy M. Schraw:** conceptualization, data curation, formal analysis, funding acquisition, investigation, methodology, project administration, resources, software, visualization, writing − original draft. **Rutu A. Rathod:** data curation, methodology, software, validation, writing − review and editing. **Amy E. Hughes:** data curation, methodology, software, validation, writing − review and editing, project administration. **Sandi L. Pruitt:** conceptualization, funding acquisition, resources, writing − review and editing. **Russell S. Kirby:** supervision, writing − review and editing. **Gary M. Shaw:** conceptualization, funding acquisition, supervision, writing − review and editing. **Philip J. Lupo:** conceptualization, funding acquisition, supervision, writing − review and editing.

## Funding

This work was supported by the National Institute on Minority Health and Health Disparities (1R01MD018577) and the National Cancer Institute (1R01CA237540). These sponsors had no active involvement in the project or in the decision to publish. The contents do not necessarily represent the official views of, nor an endorsement by the National Institutes of Health or the Texas Department of State Health Services.

## Ethics Statement

This study was approved by the Institutional Review Boards of Baylor College of Medicine and the Texas Department of State Health Services.

## Conflicts of Interest

Russell S. Kirby serves as an Editor‐in‐Chief of Birth Defects Research. Jeremy M. Schraw serves as an Associate Editor for Birth Defects Research. Gary M. Shaw has resigned as an Associate Editor for Birth Defects Research. Unrelated to this work, Sandi L. Pruitt declares consulting fees from Pfizer and Gilead. The other authors declare no conflicts of interest.

## Supporting information


**Table S1:** Distribution of Hispanic ethnic enclave index and Yost socioeconomic index scores among livebirths and cases with congenital anomalies, Texas, 1999–2018.

**Table S2:** Characteristics of women by pregnancy outcome and availability of data on residential address at delivery or termination.

**Table S3:** Associations of congenital anomalies with residence in a Hispanic/Latino ethnic enclave and/or neighborhood with low socioeconomic status in a 60% random subsample (‘discovery partition’) of births and cases with anomalies, Texas, 1999–2018.
**Table S4:** Associations of selected congenital anomalies with residence in a Hispanic/Latino ethnic enclave and/or neighborhood with low socioeconomic status.
**Table S5:** Associations of selected congenital anomalies with residence in a Hispanic/Latino ethnic enclave and/or neighborhood with low socioeconomic status.

**Table S6:** Associations of residence in a Hispanic/Latino ethnic enclave and/or neighborhood with low socioeconomic status with severe microcephaly, defined as head circumference < 3rd percentile for sex and gestational age, Texas, 1999–2018.

**Table S7:** Associations of selected congenital anomalies with residence in a Hispanic/Latino ethnic enclave and/or neighborhood with low socioeconomic status among offspring of nulliparous women, Texas, 1999–2018.

## Data Availability

Deidentified individual participant data will not be made available because the investigators are prohibited from sharing them publicly by the terms of the data use agreement. The relevant data may be obtained by application to the Texas Birth Defects Registry and Texas Department of State Health Services Institutional Review Board.
