# Supplementary material for: Neighborhood Economic Deprivation, Hispanic Ethnic Enclaves, and Congenital Anomalies in Texas
Source: Birth Defects Res. 2026 Aug 31;118(9):e70114. doi: 10.1002/bdr2.70114 (PMC13530317; doi:10.1002/bdr2.70114)
Supplement: Supplementary file 1 [file BDR2-118-e70114-s002.docx]

**Supplemental Table 2.** Characteristics of women by pregnancy outcome and availability of data on residential address at delivery or termination.

|  | **Included births**  **No. (%)** | **Births without residential data**  **No. (%)** | **Included cases**  **No. (%)** | **Cases without residential data**  **No. (%)** |
| --- | --- | --- | --- | --- |
| **TOTAL** | 7,250,822 | 447,091 | 332,935 | 22,209 |
| **Offspring sex** |  |  |  |  |
| Male | 3,705,613 (51.1) | 228,364 (51.1%) | 200,141 (60.1) | 12,378 (55.7) |
| Female | 3,545,209 (48.9) | 218,727 (48.9%) | 132,581 (39.8) | 9,014 (40.6) |
| Unknown | 0 (0.0) | 0 (0.0) | 213 (0.1) | 817 (3.7) |
| **Maternal race/ethnicity** |  |  |  |  |
| Hispanic/Latina | 3,476,679 (47.9) | 234,139 (52.4) | 157,021 (47.2) | 11,209 (50.5) |
| Non-Hispanic Black | 848,887 (11.7) | 29,860 (6.7) | 39,935 (12.0) | 1,745 (7.9) |
| Non-Hispanic White | 2,535,451 (35.0) | 173,698 (38.9) | 119.279 (35.8) | 8,504 (38.3) |
| Other non-Hispanic | 381,533 (5.3) | 8,561 (1.9) | 16,660 (5.0) | 560 (2.5) |
| Unknown | 8,272 (0.1) | 833 (0.2) | 40 (0.0) | 191 (0.9) |
| **Maternal age (years)** |  |  |  |  |
| <20 | 842,889 (11.6) | 70,901 (15.9) | 35,981 (10.8) | 3,090 (13.9) |
| 20-24 | 1,893,220 (26.1) | 137,894 (30.8) | 81,367 (24.4) | 6,132 (27.6) |
| 25-29 | 1,999,062 (27.6) | 120,119 (26.9) | 88,262 (26.5) | 5,673 (25.5) |
| 30-34 | 1,607,008 (22.2) | 77,886 (17.4) | 75,417 (22.7) | 4,042 (18.2) |
| 35-39 | 745,103 (10.3) | 32,884 (7.4) | 40,549 (12.2) | 2,361 (10.6) |
| ≥40 | 163,183 (2.3) | 7,158 (1.6) | 11,359 (3.4) | 880 (4.0) |
| Unknown | 352 (0.0) | 249 (0.1) | 0 (0.0) | 31 (0.1) |
| **Maternal education** |  |  |  |  |
| No high school diploma or equivalent | 1,855,327 (25.6) | 156,199 (34.9) | 81,614 (24.5) | 5,708 (25.7) |
| High school diploma or equivalent | 2,015,116 (27.8) | 142,077 (31.8) | 94,700 (28.4) | 5,597 (25.2) |
| Any post-high school education | 3,339,842 (46.1) | 144,540 (32.3) | 154,880 (46.5) | 5,693 (25.6) |
| Unknown | 40,537 (0.6) | 4,275 (1.0) | 1,741 (0.5) | 5,211 (23.5) |
| **Number of previous livebirths** |  |  |  |  |
| 0 | 2,753,218 (38.0) | 161,671 (36.2) | 132,759 (39.9) | 6,461 (29.1) |
| 1 | 2,233,977 (30.8) | 131,854 (29.5) | 96,849 (29.1) | 4,753 (21.4) |
| 2 | 1,295,873 (17.9) | 82,776 (18.5) | 57,631 (17.3) | 3,066 (13.8) |
| ≥3 | 885,393 (12.2) | 59,938 (13.4) | 42,825 (12.9) | 2,511 (11.3) |
| Unknown | 82,361 (1.1) | 10,852 (2.4) | 2,871 (0.9) | 5,418 (24.4) |
| **Pre-pregnancy BMI category^a^** |  |  |  |  |
| Underweight (<18.5) | 207,113 (2.9) | 9,567 (2.1) | 10,033 (3.0) | 397 (1.8) |
| Normal weight (18.5-24.9) | 2,451,992 (33.8) | 97,639 (21.8) | 118,424 (35.6) | 4,125 (18.6) |
| Overweight (25-29.9) | 1,336,470 (18.4) | 56,381 (12.6) | 65,875 (19.8) | 2,421 (10.9) |
| Obese (≥30) | 1,240,651 (17.1) | 53,241 (11.9) | 69,197 (20.8) | 2,694 (12.1) |
| Unknown | 2,014,596 (27.8) | 230,263 (51.3) | 69,406 (20.8) | 12,572 (56.6) |

Abbreviations: BMI=body mass index; nSES=neighborhood socioeconomic status.

Column percentages are presented.

a. Data were not collected prior to 2005. BMI was classified as unknown among cases and births delivered between January 1, 1999-December 31, 2004.
