# Supplementary material for: Neighborhood Economic Deprivation, Hispanic Ethnic Enclaves, and Congenital Anomalies in Texas
Source: Birth Defects Res. 2026 Aug 31;118(9):e70114. doi: 10.1002/bdr2.70114 (PMC13530317; doi:10.1002/bdr2.70114)
Supplement: Supplementary file 2 [file BDR2-118-e70114-s003.docx]

**Supplemental Table 7.** Associations of selected congenital anomalies with residence in a Hispanic/Latino ethnic enclave and/or neighborhood with low socioeconomic status among offspring of nulliparous women, Texas, 1999-2018.

|  | **Cases, No.** | **Prevalence^a^** | **aPR (95% CI)^b^** | **aPR (95% CI)^c^** |
| --- | --- | --- | --- | --- |
| **Any monitored congenital anomaly** |  |  |  |  |
| High nSES/non-enclave | 51,998 | 491.6 | 1.00 | 1.00 |
| High nSES/enclave | 6,283 | 464.7 | 0.95 (0.93-0.98) | 0.96 (0.94-0.99) |
| Low nSES/non-enclave | 33,648 | 480.0 | 1.00 (0.98-1.01) | 0.99 (0.98-1.01) |
| Low nSES/enclave | 40,810 | 475.2 | 0.99 (0.98-1.01) | 1.01 (1.00-1.03) |
| **Microcephaly** |  |  |  |  |
| High nSES/non-enclave | 1,250 | 11.8 | 1.00 | 1.00 |
| High nSES/enclave | 181 | 13.4 | 1.06 (0.90-1.24) | 1.04 (0.88-1.21) |
| Low nSES/non-enclave | 1,073 | 15.3 | 1.16 (1.06-1.26) | 1.10 (1.01-1.20) |
| Low nSES/enclave | 1,456 | 17.0 | 1.22 (1.12-1.33) | 1.17 (1.07-1.28) |
| **Other specified brain anomalies^d^** |  |  |  |  |
| High nSES/non-enclave | 2,803 | 26.5 | 1.00 | 1.00 |
| High nSES/enclave | 315 | 23.3 | 0.89 (0.79-1.00) | 0.93 (0.83-1.05) |
| Low nSES/non-enclave | 1,604 | 22.9 | 0.90 (0.84-0.96) | 0.90 (0.84-0.96) |
| Low nSES/enclave | 1,736 | 20.2 | 0.82 (0.77-0.88) | 0.88 (0.82-0.95) |
| **Unspecified facial anomalies** |  |  |  |  |
| High nSES/non-enclave | 873 | 8.3 | 1.00 | 1.00 |
| High nSES/enclave | 123 | 9.1 | 1.06 (0.88-1.29) | 0.97 (0.75-1.26) |
| Low nSES/non-enclave | 577 | 8.2 | 1.02 (0.91-1.14) | 0.98 (0.81-1.18) |
| Low nSES/enclave | 859 | 10.0 | 1.17 (1.06-1.31) | 1.10 (0.95-1.29) |
| **Ventricular septal defect** |  |  |  |  |
| High nSES/non-enclave | 6,037 | 57.1 | 1.00 | 1.00 |
| High nSES/enclave | 821 | 60.7 | 1.07 (1.00-1.15) | 1.03 (0.95-1.10) |
| Low nSES/non-enclave | 3,819 | 54.4 | 0.98 (0.94-1.02) | 0.98 (0.94-1.02) |
| Low nSES/enclave | 5,578 | 65.0 | 1.17 (1.12-1.22) | 1.10 (1.05-1.15) |
| **Atrial septal defect** |  |  |  |  |
| High nSES/non-enclave | 11,958 | 113.0 | 1.00 | 1.00 |
| High nSES/enclave | 1,528 | 113.0 | 1.01 (0.95-1.06) | 0.98 (0.93-1.03) |
| Low nSES/non-enclave | 8,761 | 124.9 | 1.10 (1.07-1.14) | 1.10 (1.06-1.13) |
| Low nSES/enclave | 11,281 | 131.4 | 1.17 (1.14-1.21) | 1.13 (1.09-1.16) |
| **Pulmonary valve anomalies** |  |  |  |  |
| High nSES/non-enclave | 1,091 | 10.3 | 1.00 | 1.00 |
| High nSES/enclave | 181 | 13.4 | 1.31 (1.12-1.54) | 1.30 (1.11-1.53) |
| Low nSES/non-enclave | 863 | 12.3 | 1.21 (1.10-1.33) | 1.18 (1.08-1.30) |
| Low nSES/enclave | 1,087 | 12.7 | 1.26 (1.15-1.39) | 1.24 (1.13-1.37) |
| **Other specified cardiac anomalies^e^** |  |  |  |  |
| High nSES/non-enclave | 1,492 | 14.1 | 1.00 | 1.00 |
| High nSES/enclave | 203 | 15.0 | 1.07 (0.92-1.24) | 1.00 (0.86-1.16) |
| Low nSES/non-enclave | 1,257 | 17.9 | 1.27 (1.17-1.38) | 1.24 (1.14-1.34) |
| Low nSES/enclave | 1,843 | 21.5 | 1.54 (1.42-1.66) | 1.40 (1.29-1.51) |
| **Patent ductus arteriosus** |  |  |  |  |
| High nSES/non-enclave | 5,652 | 53.4 | 1.00 | 1.00 |
| High nSES/enclave | 755 | 55.8 | 1.05 (0.98-1.14) | 1.02 (0.94-1.10) |
| Low nSES/non-enclave | 3,987 | 56.8 | 1.09 (1.04-1.13) | 1.07 (1.03-1.12) |
| Low nSES/enclave | 5,429 | 63.2 | 1.21 (1.17-1.27) | 1.15 (1.10-1.20) |
| **Pulmonary artery anomalies** |  |  |  |  |
| High nSES/non-enclave | 1,717 | 16.2 | 1.00 | 1.00 |
| High nSES/enclave | 350 | 25.9 | 1.59 (1.42-1.79) | 1.40 (1.25-1.58) |
| Low nSES/non-enclave | 1,595 | 22.7 | 1.36 (1.26-1.46) | 1.32 (1.23-1.42) |
| Low nSES/enclave | 2,790 | 32.5 | 1.95 (1.83-2.09) | 1.63 (1.52-1.75) |
| **Other aorta anomalies^f^** |  |  |  |  |
| High nSES/non-enclave | 1,429 | 13.5 | 1.00 | 1.00 |
| High nSES/enclave | 202 | 14.9 | 1.13 (0.97-1.31) | 1.12 (0.97-1.31) |
| Low nSES/non-enclave | 970 | 13.8 | 1.07 (0.98-1.16) | 1.07 (0.98-1.16) |
| Low nSES/enclave | 1,291 | 15.0 | 1.19 (1.09-1.29) | 1.18 (1.08-1.30) |
| **Aortic valve insufficiency** |  |  |  |  |
| High nSES/non-enclave | 799 | 7.6 | 1.00 | 1.00 |
| High nSES/enclave | 87 | 6.4 | 0.85 (0.68-1.06) | 0.90 (0.66-1.22) |
| Low nSES/non-enclave | 596 | 8.5 | 1.14 (1.02-1.27) | 1.19 (0.97-1.45) |
| Low nSES/enclave | 678 | 7.9 | 1.07 (0.96-1.19) | 1.12 (0.94-1.33) |
| **Anomalies of the great veins** |  |  |  |  |
| High nSES/non-enclave | 658 | 6.2 | 1.00 | 1.00 |
| High nSES/enclave | 98 | 7.2 | 1.14 (0.93-1.42) | 1.24 (0.92-1.67) |
| Low nSES/non-enclave | 497 | 7.1 | 1.12 (0.99-1.26) | 1.23 (0.99-1.52) |
| Low nSES/enclave | 633 | 7.4 | 1.14 (1.02-1.29) | 1.24 (1.03-1.49) |
| **Pyloric stenosis** |  |  |  |  |
| High nSES/non-enclave | 1,593 | 15.1 | 1.00 | 1.00 |
| High nSES/enclave | 200 | 14.8 | 0.93 (0.80-1.08) | 0.92 (0.79-1.07) |
| Low nSES/non-enclave | 1,349 | 19.2 | 1.07 (0.99-1.16) | 1.12 (1.03-1.20) |
| Low nSES/enclave | 1,634 | 19.0 | 1.03 (0.96-1.11) | 1.02 (0.94-1.11) |
| **Hirschsprung disease** |  |  |  |  |
| High nSES/non-enclave | 169 | 1.6 | 1.00 | 1.00 |
| High nSES/enclave | 18 | 1.3 | 0.82 (0.51-1.34) | 0.81 (0.37-1.79) |
| Low nSES/non-enclave | 111 | 1.6 | 0.97 (0.76-1.24) | 0.54 (0.29-0.99) |
| Low nSES/enclave | 102 | 1.2 | 0.73 (0.56-0.94) | 0.66 (0.42-1.04) |
| **Female genital anomalies** |  |  |  |  |
| High nSES/non-enclave | 927 | 8.8 | 1.00 | 1.00 |
| High nSES/enclave | 163 | 12.1 | 1.31 (1.11-1.55) | 1.14 (0.97-1.36) |
| Low nSES/non-enclave | 630 | 9.0 | 1.01 (0.91-1.12) | 0.98 (0.88-1.09) |
| Low nSES/enclave | 1,205 | 14.0 | 1.50 (1.36-1.65) | 1.24 (1.12-1.37) |
| **Undescended testicle** |  |  |  |  |
| High nSES/non-enclave | 2,728 | 25.8 | 1.00 | 1.00 |
| High nSES/enclave | 339 | 25.1 | 0.97 (0.86-1.08) | 0.95 (0.85-1.06) |
| Low nSES/non-enclave | 1,802 | 25.7 | 1.00 (0.94-1.06) | 0.98 (0.92-1.04) |
| Low nSES/enclave | 2,393 | 27.9 | 1.08 (1.02-1.15) | 1.05 (0.98-1.12) |
| **Hypospadias, epispadias, chordee** |  |  |  |  |
| High nSES/non-enclave | 6,212 | 58.7 | 1.00 | 1.00 |
| High nSES/enclave | 574 | 42.5 | 0.76 (0.70-0.83) | 0.86 (0.79-0.94) |
| Low nSES/non-enclave | 3,507 | 50.0 | 0.95 (0.91-0.99) | 0.95 (0.91-0.99) |
| Low nSES/enclave | 2,904 | 33.8 | 0.68 (0.65-0.71) | 0.82 (0.78-0.87) |
| **Other male genital anomalies^g^** |  |  |  |  |
| High nSES/non-enclave | 2,963 | 28.0 | 1.00 | 1.00 |
| High nSES/enclave | 287 | 21.2 | 0.78 (0.69-0.88) | 0.84 (0.74-0.94) |
| Low nSES/non-enclave | 1,591 | 22.7 | 0.89 (0.83-0.95) | 0.87 (0.82-0.93) |
| Low nSES/enclave | 1,469 | 17.1 | 0.70 (0.65-0.75) | 0.77 (0.71-0.82) |
| **Other specified ureter anomalies^h^** |  |  |  |  |
| High nSES/non-enclave | 1,139 | 10.8 | 1.00 | 1.00 |
| High nSES/enclave | 121 | 8.9 | 0.86 (0.71-1.03) | 0.90 (0.68-1.18) |
| Low nSES/non-enclave | 670 | 9.6 | 0.94 (0.85-1.04) | 0.94 (0.77-1.13) |
| Low nSES/enclave | 741 | 8.6 | 0.87 (0.79-0.97) | 0.92 (0.78-1.07) |
| **Skull, face, and jaw anomalies^i^** |  |  |  |  |
| High nSES/non-enclave | 3,472 | 32.8 | 1.00 | 1.00 |
| High nSES/enclave | 414 | 30.6 | 0.94 (0.85-1.04) | 0.95 (0.86-1.06) |
| Low nSES/non-enclave | 1,766 | 25.2 | 0.85 (0.80-0.90) | 0.88 (0.83-0.94) |
| Low nSES/enclave | 2,407 | 28.0 | 0.95 (0.90-1.01) | 0.98 (0.92-1.04) |
| **Other foot anomalies^j^** |  |  |  |  |
| High nSES/non-enclave | 1,910 | 18.1 | 1.00 | 1.00 |
| High nSES/enclave | 263 | 19.5 | 1.07 (0.94-1.22) | 1.08 (0.95-1.23) |
| Low nSES/non-enclave | 1,277 | 18.2 | 1.01 (0.94-1.09) | 1.01 (0.94-1.09) |
| Low nSES/enclave | 1,664 | 19.4 | 1.07 (0.99-1.15) | 1.08 (1.00-1.17) |
| **Polydactyly** |  |  |  |  |
| High nSES/non-enclave | 2,176 | 20.6 | 1.00 | 1.00 |
| High nSES/enclave | 254 | 18.8 | 0.91 (0.80-1.04) | 0.78 (0.63-0.96) |
| Low nSES/non-enclave | 1,756 | 25.0 | 1.16 (1.08-1.24) | 1.02 (0.89-1.17) |
| Low nSES/enclave | 1,898 | 22.1 | 1.04 (0.97-1.12) | 0.87 (0.78-0.98) |
| **Down syndrome** |  |  |  |  |
| High nSES/non-enclave | 996 | 9.4 | 1.00 | 1.00 |
| High nSES/enclave | 155 | 11.5 | 1.24 (1.05-1.47) | 1.13 (0.95-1.34) |
| Low nSES/non-enclave | 566 | 8.1 | 1.08 (0.97-1.21) | 1.06 (0.95-1.19) |
| Low nSES/enclave | 813 | 9.5 | 1.22 (1.10-1.36) | 1.06 (0.95-1.19) |

a) Per 10,000 livebirths.
b) Adjusted for year of delivery, maternal age (<20, 20-24, 25-29, 30-34, 35-39, ≥40 years), education (no high school diploma or equivalent, high school diploma or equivalent, any higher education or vocational training), number of previous livebirths (none, one, two, three or more), and pre-pregnancy body mass index category (<18.5, 18.5-24.9, 25-29.9, ≥30, unknown). Relative to a referent category of individuals residing in high nSES/non-enclave neighborhoods.
c) Adjusted for the variables in (b.), plus maternal race (Black, White, other, or unknown) and ethnicity (Hispanic/Latina or non-Hispanic/Latina).
d) Includes: enlarged brain and/or head; megalencephaly, macrocephaly, or porencephaly; porencephalic, cerebral, ventricular, or choroid plexus cysts; cortical or cerebral atrophy; cranial nerve defects, brainstem anomalies, Arnold-Chiari malformation without spina bifida; and cortical dysplasia (cerebral).
e) Includes: dextrocardia without situs inversus; cor triatriatum; pulmonary infundibular (subvalvular) stenosis; trilogy of Fallot; pericardium or myocardium anomalies; cardiomegaly and cardiomyopathy; rhabdomyoma or tumor of the heart; ventricular hypertrophy or ventricular septal thickening; congenital heart block; ectopia cordis; Shone’s complex; hypoplastic heart, right heart, left ventricle, ventricle not otherwise specified, or artium; long Q-T syndrome; right or left ventricular outflow tract obstruction not otherwise specified; Uhl's disease; anomalies of coronary artery or sinus anomalies; dilated coronary sinus; and other defects of the atria.
f) Includes: absence, hypoplasia, atresia, stenosis, atrophy, elongation, or enlargement of aorta; pseudotruncus arteriosus; proximal distal transverse arch hypoplasia; narrow aortic isthmus, hypoplastic or interrupted aortic arch; supra-aortic stenosis (supravalvular); persistent right aortic arch; aneurysm of sinus of Valsalva; vascular ring (aorta); double aortic arch; overriding aorta; dextroposition or malalignment of aorta; congenital aneurysm or dilatation of aorta; collateral vessel involving aorta; and pseudocoarctation of aorta.
g) Includes absence of testis; monorchidism; aplasia or hypoplasia of testis or scrotum; polyorchidism; bifid scrotum; anomalies of the vas deferens; and micro- or macro-genitalia.
h) Includes: absence of the ureter, accessory ureter, ectopic ureter, ureterocele, short ureter, and variations of vesicoureteral reflux.
i) Includes: asymmetry of the face, heard or skull; dolicocephaly; plagiocephaly; scaphocephaly, trigonocephaly, and other skull deformities without mention of craniosynostosis.
j) Includes: pes cavus; claw foot; short Achilles tendon; equinus foot; other specified deformities of the ankle and/or toes; dorsiflexion of foot; upturned foot; hyperextended foot; and talipes, clubfoot, positional defects, or deformities of the foot not otherwise specified.
