# Supplementary material for: Neighborhood Economic Deprivation, Hispanic Ethnic Enclaves, and Congenital Anomalies in Texas
Source: Birth Defects Res. 2026 Aug 31;118(9):e70114. doi: 10.1002/bdr2.70114 (PMC13530317; doi:10.1002/bdr2.70114)
Supplement: Supplementary file 3 [file BDR2-118-e70114-s004.docx]

**Supplemental Table 1.** Distribution of Hispanic ethnic enclave index and Yost socioeconomic index scores among livebirths and cases with congenital anomalies, Texas, 1999-2018.

|  | **Livebirths, No. (%)** | **Cases, No. (%)** |
| --- | --- | --- |
| **TOTAL** | 7,250,822 (100.0) | 332,935 (100.0) |
| **Hispanic ethnic enclave index score** |  |  |
| Quintile 1 (lowest) | 998,312 (13.8) | 45,316 (13.6) |
| Quintile 2 | 1,300,391 (17.9) | 59,776 (18.0) |
| Quintile 3 | 1,429,339 (19.7) | 66,714 (20.0) |
| Quintile 4 | 687,493 (9.5) | 31,699 (9.5) |
| Quintile 5 (highest) | 2,835,287 (39.1) | 129,430 (38.9) |
| **Yost socioeconomic index score** |  |  |
| Quintile 1 (lowest) | 1,550,753 (21.4) | 71,526 (21.5) |
| Quintile 2 | 1,467,103 (20.2) | 67,360 (20.2) |
| Quintile 3 | 1,336,239 (18.4) | 60,913 (18.3) |
| Quintile 4 | 1,470,875 (20.3) | 67,865 (20.4) |
| Quintile 5 (highest) | 1,425,872 (19.7) | 65,271 (19.6) |
