# Supplementary material for: Neighborhood Economic Deprivation, Hispanic Ethnic Enclaves, and Congenital Anomalies in Texas
Source: Birth Defects Res. 2026 Aug 31;118(9):e70114. doi: 10.1002/bdr2.70114 (PMC13530317; doi:10.1002/bdr2.70114)
Supplement: Supplementary file 4 [file BDR2-118-e70114-s005.docx]

**Supplemental Table 6.** Associations of residence in a Hispanic/Latino ethnic enclave and/or neighborhood with low socioeconomic status with severe microcephaly, defined as head circumference <3^rd^ percentile for sex and gestational age, Texas, 1999-2018.

|  | **Cases, No.** | **Prevalence^a^** | **aPR (95% CI)^b^** | **aPR (95% CI)^c^** |
| --- | --- | --- | --- | --- |
| **Microcephaly** |  |  |  |  |
| High nSES-non-enclave | 933 | 3.6 | 1.00 | 1.00 |
| High nSES-enclave | 142 | 4.2 | 1.07 (0.89-1.27) | 1.09 (0.91-1.31) |
| Low nSES-non-enclave | 972 | 5.2 | 1.27 (1.15-1.40) | 1.28 (1.16-1.41) |
| Low nSES-enclave | 1,395 | 5.6 | 1.29 (1.17-1.41) | 1.37 (1.25-1.51) |

Abbreviations: aPR=adjusted prevalence ratio; CI=confidence interval; nSES=neighborhood socioeconomic status.

a) Per 10,000 livebirths.
b) Adjusted for year of delivery, maternal age (<20, 20-24, 25-29, 30-34, 35-39, ≥40 years), education (no high school diploma or equivalent, high school diploma or equivalent, any higher education or vocational training), number of previous livebirths (none, one, two, three or more), and pre-pregnancy body mass index category (<18.5, 18.5-24.9, 25-29.9, ≥30, unknown). Relative to a referent category of individuals residing in high nSES/non-enclave neighborhoods.
c) Adjusted for the variables listed above, plus maternal race (Black, White, other, or unknown) and ethnicity (Hispanic/Latina or non-Hispanic/Latina).
